# Serum Calcium Level as a Useful Surrogate for Risk of Elevated Intraocular Pressure

**DOI:** 10.3390/jcm10091839

**Published:** 2021-04-23

**Authors:** Yu-Min Chang, Jiann-Torng Chen, Ming-Cheng Tai, Wei-Liang Chen, Ying-Jen Chen

**Affiliations:** 1 Department of Ophthalmology, Tri-Service General Hospital, School of Medicine, National Defense Medical Center, Taipei 114, Taiwan; m7886916@yahoo.com.tw (Y.-M.C.); jt66chen@gmail.com (J.-T.C.); mingtai1966@yahoo.com.tw (M.-C.T.); 2 Division of Family Medicine, Department of Family and Community Medicine, Tri-Service General Hospital, School of Medicine, National Defense Medical Center, Taipei 114, Taiwan; weiliang0508@gmail.com; 3 Division of Geriatric Medicine, Department of Family and Community Medicine, Tri-Service General Hospital, School of Medicine, National Defense Medical Center, Taipei 114, Taiwan

**Keywords:** intraocular pressure, serum calcium, female

## Abstract

Background: Uncontrolled intraocular pressure (IOP) plays a principal role in the deterioration of glaucoma, and the intraocular pressure is also accepted as the most important modifiable factor. Calcium ion has been found to play a vital role in regulating the resistance of the trabecular meshwork in humans. However, the relationship between serum total calcium and IOP has not been well-established. Methods: We investigated the association between serum total calcium and the IOP in a large population (14,037 eligible participants, consisting of 7712 men and 6325 women, were included) at the Tri-Service General Hospital from 2010 to 2016. Several models of covariate adjustments associated with IOP were designed. Univariate and multivariate regression analysis was performed for gender differences in the association between the serum total calcium level and IOP. Results: There was a significant relationship between serum total calcium levels and IOP in women and men with a β coefficient of 0.050 (95% confidence interval (CI), 0.030–0.069) and 0.025 (95%CI, 0.007–0.043). Notably, participants in the highest tertiles of serum total calcium levels had significantly higher IOP, in both the male and female participants. Conclusions: Our study shows that IOP is significantly associated with serum total calcium levels in a large Asian population. This study supports the notion that serum total calcium may play an important role in groups at high risk for elevated IOP.

## 1. Introduction

Glaucoma is a chronic and irreversible disease characterized by progressive loss of retinal ganglion cells [1]; it is a leading cause of permanent blindness in the world. Intraocular pressure (IOP) has been well accepted as the most important modifiable factor for development of glaucoma and the goal of most therapy is to control IOP. Many studies have reported positive associations between IOP and several cardiometabolic conditions, including hypertension, diabetes [2], postprandial glucose [3], coronary atherosclerosis [4], and obesity [5].

The aqueous humor (AH) drainage system in human eyes has two pathways, including conventional or trabecular outflow and uveoscleral outflow. The trabecular outflow is the main drainage route in humans, which is composed of the trabecular meshwork, the juxtacanalicular connective tissue, the endothelial lining of Schlemm’s canal, the collecting channels, and the aqueous veins [6]. If any structure in the trabecular outflow pathway is damaged, AH drainage can be impaired, which causes elevation of IOP.

Calcium ion plays an important role in cell signaling and cell contraction; its concentration is modulated by many factors, including the cellular environment. Dysregulation of calcium homeostasis has been found in many neurodegenerative diseases, such as Huntington’s disease, Parkinson’s disease, Alzheimer’s disease, amyotrophic lateral sclerosis, and multiple sclerosis [7,8]. In a primary open angle glaucoma (POAG) model from postmortem donor eyes, one study discovered the mitochondrial function of trabecular meshwork (TM) cells was damaged, which caused the cells to be abnormally vulnerable to calcium ion stress. Hence, the IOP is uncontrolled because of dysfunction in calcium regulation in these cells [9]. Another study reports that transient receptor potential vanilloid 4 (TRPV4) is a central channel for calcium ion and mechanical stretch-sensitivity in human TM cells. Mechanical stress, such as swelling and pressure, can activate TRPV4 and cause extracellular matrix (ECM) remodeling associated with increased TM stiffness and contractility. Finally, when the TM outflow is obstructed, the IOP is elevated [10].

However, serum total calcium is now believed to be associated with metabolic syndrome and other cardiometabolic disease [11,12]. The relationship between metabolic syndrome and high IOP was discovered in previous studies [5,13]. To date, no cross-sectional studies have investigated the association between serum total calcium and IOP in an Asian population with a large sample size. Hence, the aim of our study is to explore the influence of serum total calcium on IOP in an Asian population.

## 2. Materials and Methods

### 2.1. Design of the Study

**Figure 1:**
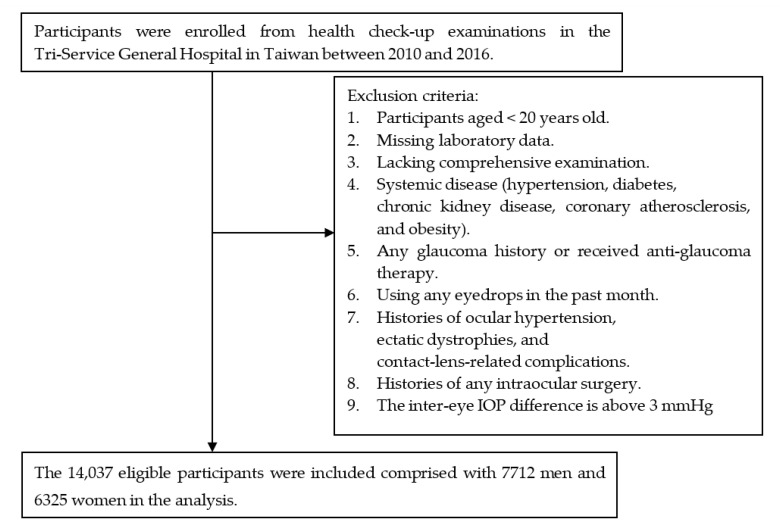
Flowchart of subject selection.

We collected the medical records of healthy examinations including laboratory examinations, ophthalmological examinations, body composition, and self-reported questionnaires between 2010 and 2016 in a medical center, the Tri-Service General Hospital (TSGH) in Taiwan. Only participants over 20 years old were included in this study. This was a retrospective study to determine the association of IOP with serum total calcium levels. Our exclusion criteria were as follows: missing laboratory data; those lacking comprehensive examinations; any systemic disease that could affect the IOP and homeostasis of serum calcium (hypertension, diabetes, chronic kidney disease, coronary atherosclerosis, and obesity); any glaucoma history or having received anti-glaucoma therapy; using any eyedrops in the past month; histories of ocular hypertension, ectatic dystrophies, and contact-lens-related complications; histories of any intraocular surgery; and an inter-eye IOP difference above 3 mmHg [14]. If the participants received more than one healthy examination during this period, we only selected the data at the first visit to analyze. Finally, 14,037 eligible participants, consisting of 7712 men and 6325 women, were included in the analysis (Figure 1). The study was conducted in accordance with the principles of the Declaration of Helsinki, and it received institutional review board approval from TSGH.

### 2.2. Ophthalmological Examinations

Ophthalmological examinations were performed by professional ophthalmologists in a standard ophthalmological examination room in TSGH, including best-corrected visual acuity (BCVA), IOP, biomicroscopic examinations, and dilated fundus examinations. A CT-80 non-contact computerized tonometer was used to obtain the IOP of both eyes, and the mean IOP values of both eyes were recorded for logistic regression.

### 2.3. Covariates

Age, gender, and personal history (smoking and drinking) were obtained by a self-assessment questionnaire. Bioelectrical impedance analysis (InBody720, Biospace, Inc., Cerritos, CA, USA) was applied to measure the percentage body fat. Participants must have fasted for at least 8 h before blood draws. Uric acid, serum total cholesterol, aspartate aminotransferase, creatinine, highly sensitive C-reactive protein, thyroid-stimulating hormone, and total calcium were included in laboratory examinations and were analyzed by various methods. A Hitachi 737 automated analyzer was used to measure uric acid. Enzymatic colorimetric methods were applied to detect total cholesterol and aspartate aminotransferase. The Jaffe method using alkaline picrate was used to measure creatinine. Highly sensitive C-reactive protein was accessed by latex-enhanced nephelometry. An immune-enzymatic assay was applied to detect thyroid-stimulating hormone. Finally, the o-cresolphthalein complexone method was used to measure serum total calcium levels. All methods were executed based on the relevant guidelines and regulations of TSGH.

### 2.4. Statistical Analyses

The differences between men and women with respect to demographic characteristics and laboratory data were analyzed by *t*-test and chi-squared test. Gender difference in IOP was found in many studies. Some studies revealed the IOP was higher in the male group than in the female group [15,16]. However, some studies proposed the opposite view [17,18]. The effect of modification by serum total calcium level and gender was tested by interaction terms in the models for the IOP. There were significant interactions between serum total calcium level and gender. According to the significant findings of the interaction testing, further stratified analyses were performed. Multivariate linear and logistic regression models were used to investigate the association between serum total calcium and IOP. Three models of covariate adjustments were designed: Model 1 = unadjusted; Model 2 = Model 1 + age, gender, percentage body fat, total cholesterol, uric acid, aspartate aminotransferase, creatinine, highly sensitive C-reactive protein, and thyroid-stimulating hormone, which were recognized as correlated variables with IOP [19,20,21]; and Model 3 = Model 2 + smoking and drinking, which were also recognized as correlated variables with IOP [22]. Multivariate linear regression analysis was used for gender differences in the association between the serum total calcium level and IOP. In addition, we divided serum total calcium levels into tertiles to perform tertiles-based analysis, and participants in the lowest tertile were regarded as the reference group. The cut-off levels of serum total calcium for the tertiles were as follows: 5 mg/dL < T1 ≤ 9.1 mg/dL; 9.1 mg/dL < T2 ≤ 9.4 mg/dL; and 9.4 mg/dL < T3 ≤ 12 mg/dL. We also used the logistic regression to calculate the odds ratios and to investigate the relationship between serum total calcium and the risk of high IOP. In our study, high IOP was defined as 18 mmHg, according to a previous study [23]. A receiving operating characteristic (ROC) curve plot was used to find the optimal cut-off of serum total calcium. Furthermore, the area under the ROC (AUROC) and the corresponding 95% confidence intervals (CI) were all calculated. A P value less than 0.05 was defined as statistically significant for all analyses. Data analysis of this study was conducted using IBM Statistical Product and Service Solutions Statistics version 22.0.

## 3. Results

### 3.1. Demographics of the Participants

Clinical demographic information including age, IOP, percentage body fat, and biochemical data in men and women are presented in Table 1. The study group comprised 14,037 participants (7712 men and 6325 women; mean age 46.88 ± 13 years and 47.00 ± 12.61 years, respectively). Table 1 shows that the IOP was higher in men (14.80 ± 3.10 mmHg) and that the percentage body fat (31.94 ± 6.67%) and total cholesterol (191.29 ± 36.66 mg/dL) were higher in women.

### 3.2. Association between Serum Total Calcium and Intraocular Pressure

In our study, we found a prominent relationship between serum total calcium levels and IOP. The results were analyzed by linear regression and are shown in Table 2. The β coefficient of the IOP was 0.045 (95% confidence interval, 0.033–0.058, *p* < 0.001), 0.039 (95% confidence interval, 0.026–0.053, *p* < 0.001), and 0.040 (95% confidence interval, 0.027–0.053, *p* < 0.001) in Models 1, 2, and 3, respectively. We further divided the participants into two groups by gender, and there was still a significant association between serum total calcium levels and IOP.

As shown in Table 3, serum total calcium levels were divided into tertiles to investigate the association with IOP. Positive associations were found between serum total calcium levels and IOP regardless of gender. In the male group, participants in the highest tertiles of serum total calcium levels had significantly higher IOP with a β coefficient of 0.022 (95% confidence interval, 0.005–0.039, *p* < 0.010) in Model 3 and in the female group, participants in the highest tertiles of serum total calcium levels also had significantly higher IOP with a β coefficient of 0.046 (95% confidence interval, 0.025–0.067, *p* < 0.001) in the same model.

**Figure 2:**
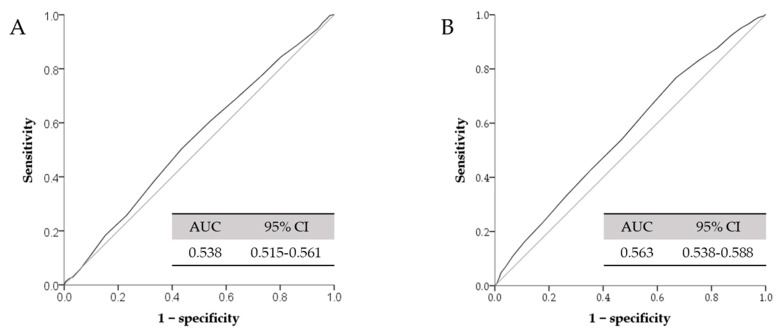
ROCs for serum total calcium and high IOP. (**A**): male group; (**B**): female group. AUC: area under the curve; ROC: receiver operating characteristic. High IOP: > 18 mmHg.

We also performed the logistic regression to examine the association between different serum total calcium tertiles and high IOP and the result is demonstrated in Table 4. In the female population, the risk of high IOP was significantly associated with the higher tertiles of serum total calcium levels in Model 1 (odds ratio = 1.599, 95% confidence interval = 1.171–2.184, *p* = 0.003), Model 2 (odds ratio = 1.522, 95% confidence interval = 1.105–2.097, *p* = 0.010), and Model 3 (odds ratio = 1.539, 95% confidence interval = 1.116–2.122, *p* = 0.008). However, the odds ratios between serum total calcium and high IOP were not significant in the male group. Figure 2 summarizes the optimal cut-off value of serum total calcium by using ROC analysis. The AUROC value was 0.538 (95% confidence interval = 0.515–0.561) in the male group and 0.563 (95% confidence interval = 0.538–0.588) in the female group. The odds ratios for developing high IOP (>18 mmHg) in different models are showed in Table 5. The optimal cut-off value of serum total calcium level was 9.35 mg/dL in the male group by using maximal Youden’s index with sensitivity and specificity (50.4/56.5%). Likewise, the optimal cut-off value of serum total calcium level was 9.05 mg/dL in the female group with sensitivity and specificity (76.8/33.0%). We found the significant occurrence of high IOP in the cut-off value in the female group. In contrast, there was no significant difference in the cut-off value in the male group.

## 4. Discussion

In the current study, we observed an association between serum total calcium and IOP. Regardless of gender, participants with higher serum total calcium levels were associated with higher IOP. Furthermore, in the female population, the risk of high IOP was significantly associated with the higher tertiles of serum total calcium levels. To our best knowledge, our study is the first cross-sectional and retrospective study to evaluate the association between serum total calcium and IOP in an Asian population.

Several lines of evidence suggest that calcium ion is a major cation that triggers a series of cascades and causes an impairment of conventional pathway outflow [9,24]. A study demonstrated that TRPV4 channels serve as important components of the mechanosensitive, calcium ion-initiated pathway within the TM and cause ECM remodeling, which regulates TM stiffness [10]. Furthermore, a study identified a new gene, Cacna2d1, which encodes the voltage-dependent calcium channel complex in the trabecular meshwork and ciliary body, and modulates the IOP [25]. However, these studies were all conducted in in vitro, not in vivo models. On the other hand, our study found positive associations between serum total calcium levels and IOP in an Asian population. This is the first study to demonstrate that serum total calcium could play a critical role in IOP modulation in humans.

Although the possible mechanism of serum total calcium and IOP is unclear, recently, more and more studies have discovered serum total calcium to have some effects on cardiometabolic diseases [11,12,13]. Disturbance of calcium homeostasis leads to insulin resistance and vascular resistance, which are the crucial factors in cardiometabolic diseases [11]. Previous studies also showed cardiometabolic diseases were associated with IOP [2,3,4,5]. Therefore, we propose that the possible mechanism of the association between serum total calcium and IOP may be insulin resistance, or other mechanisms that can cause insulin resistance [11].

IOP can be influenced by many systemic conditions, including blood pressure [26,27], fasting glucose [28], atherosclerotic diseases [26], chronic kidney disease [29], and thyroid hormone [21]. In addition, previous studies also discovered a gender difference of IOP in various populations [15,16,17,18]. In our study, the risk of high IOP was significantly associated with the higher tertiles of serum total calcium levels in the female population. The possible reasons for a gender difference might be related to the percentage body fat. Table 1 shows that the percentage body fat is significantly higher in women than in men (*p* value < 0.001). Published papers also revealed a positive correlation between IOP and obesity [5,30]. The plausible underlying mechanisms have been explained in many studies [31,32]. Excess intraorbital fat tissue can cause episcleral venous pressure increases and ultimately, outflow capability decreases. Furthermore, blood viscosity increases due to obesity and contributes to resistance in episcleral veins. We proposed that the percentage body fat may be a confounding factor regarding the relationship between IOP and serum total calcium levels. Hence, we used three different models to calibrate and reduce the bias. However, the influence of obesity on IOP is far beyond our imagination, and the hormone influence can be considered, so in Table 4 and Table 5 the significant association between developing high IOP and serum total calcium levels was only found in women.

Nevertheless, this study has some limitations in spite of our caution. First, this study employed a cross-sectional design that could not reveal causality. Longitudinal analysis is required for future specialists to explore the association between IOP and total calcium levels. Second, the IOP of both eyes was measured only once, and we used the mean IOP of both eyes for analysis. Fluctuations in IOP were ignored in our study, and the mean value of IOP may not reflect the real situation. Third, the study population was recruited from a single center and more large-scale studies from multiple centers should be considered. Fourth, the central corneal thickness (CCT) could influence the IOP measurement reported by many studies [33,34] and this could have caused the IOP measurement bias. However, according to the Singapore Malay Eye Study, Aung et al. discovered age, weight, BMI, presence of diabetes, HbA1C levels, serum glucose levels, metabolic syndrome, and CKD were significantly associated with CCT [35]. In our study, we excluded systemic and ocular diseases (hypertension, diabetes, chronic kidney disease, coronary atherosclerosis, obesity, ocular hypertension, ectatic dystrophies, contact-lens-related complications, and any intraocular surgery) to reduce the influence of CCT on the IOP. Lastly, because the participants’ diseases were obtained from self-report histories, we could not exclude the possibility of participants’ recall bias.

## 5. Conclusions

Our study highlights that IOP was significantly associated with serum total calcium levels in a large Asian population. Notably, we also found that a serum total calcium level above 9.05 mg/dL was an important risk factor to predict the high IOP in the female group. Although the exact pathophysiological mechanism underlying the association between serum total calcium and IOP is still not clear, our study provides evidence for future researchers to evaluate it in longitudinal and multi-center trials.

## Figures and Tables

**Table 1:** Characteristics of participants between gender.

Variables	Male (*n* = 7712)	Female (*n* = 6325)	*p*-Value
**Continuous Variables, Mean (SD)**			
Age (years)	46.88 (13.00)	47.00 (12.61)	0.577
IOP (mmHg)	14.80 (3.10)	14.54 (3.09)	<0.001
Percentage body fat (%)	25.00 (6.33)	31.94 (6.67)	<0.001
Total cholesterol (mg/dL)	189.31 (36.09)	191.29 (36.66)	0.001
Uric acid (mg/dL)	6.49 (1.33)	4.76 (1.10)	<0.001
Aspartate aminotransferase (U/L)	23.01 (13.52)	19.63 (9.31)	<0.001
Creatinine (mg/dL)	0.97 (0.34)	0.68 (0.17)	<0.001
highly sensitive C-reactive protein (mg/dL)	0.25 (0.56)	0.21 (0.42)	<0.001
Thyroid-stimulating hormone (IU/mL)	2.15 (1.54)	2.47 (1.96)	<0.001
Serum total calcium (mg/dL)	9.28 (0.40)	9.21 (0.41)	0.310
**Category Variables, (*n*, %)**			
Smoking	3448 (44.7)	515 (8.1)	<0.001
Drinking	4242 (55.0)	1544 (24.1)	<0.001

Abbreviations: SD, standard deviation; IOP, intraocular pressure.

**Table 2:** Association between serum total calcium and the intraocular pressure.

Variable	Model ^a^ 1 β (95% CI)	*p* Value	Model ^a^ 2 β (95% CI)	*p* Value	Model ^a^ 3 β (95% CI)	*p* Value
Total	0.045 (0.033–0.058)	<0.001	0.039 (0.026–0.053)	<0.001	0.040 (0.027–0.053)	<0.001
Male	0.037 (0.020–0.055)	<0.001	0.024 (0.007–0.042)	0.007	0.025 (0.007–0.043)	0.006
Female	0.051 (0.032–0.070)	<0.001	0.049 (0.030–0.069)	<0.001	0.050 (0.030–0.069)	<0.001

^a^ Adjusted covariates: Model 1 = unadjusted; Model 2 = Model 1 + age, gender, percentage body fat, total cholesterol, uric acid, aspartate aminotransferase, creatinine, highly sensitive C-reactive protein and thyroid-stimulating hormone; Model 3 = Model 2 + smoking and drinking. Abbreviations: CI, confidence interval.

**Table 3:** Association between tertiles of serum total calcium and intraocular pressure.

Variables	Tertiles	Model ^a^ 1 β (95% CI)	*p* Value	Model ^a^ 2 β (95% CI)	*p* Value	Model ^a^ 3 β (95% CI)	*p* Value
Total	T2 ^b^ vs. T1 ^b^	0.023 (0.011–0.036)	<0.001	0.023 (0.010–0.035)	<0.001	0.023 (0.011–0.035)	<0.001
	T3 ^b^ vs. T1 ^b^	0.039 (0.026–0.052)	<0.001	0.035 (0.022–0.048)	<0.001	0.035 (0.022–0.048)	<0.001
Male	T2 ^b^ vs. T1 ^b^	0.021 (0.005–0.038)	0.012	0.020 (0.004–0.037)	0.016	0.021 (0.004–0.037)	0.013
	T3 ^b^ vs. T1 ^b^	0.031 (0.014–0.047)	<0.001	0.022 (0.005–0.038)	0.012	0.022 (0.005–0.039)	0.010
Female	T2 ^b^ vs. T1 ^b^	0.024 (0.006–0.043)	0.011	0.023 (0.004–0.042)	0.017	0.024 (0.004–0.043)	0.016
	T3 ^b^ vs. T1 ^b^	0.048 (0.028–0.067)	<0.001	0.046 (0.025–0.066)	<0.001	0.046 (0.025–0.067)	<0.001

^a^ Adjusted covariates: Model 1 = unadjusted; Model 2 = Model 1 + age, gender, percentage body fat, total cholesterol, uric acid, aspartate aminotransferase, creatinine, highly sensitive C-reactive protein, and thyroid-stimulating hormone; Model 3 = Model 2 + smoking and drinking. ^b^ Total calcium level: T1: 5–9.1 mg/dL, T2: 9.1–9.4 mg/dL, T3: 9.4–12 mg/dL.

**Table 4:** Gender difference in association between serum total calcium tertiles and the presence of high IOP.

Variables	Tertiles	Model ^a^ 1 Odds Ratio (95% CI)	*p* Value	Model ^a^ 2 Odds Ratio (95% CI)	*p* Value	Model ^a^ 3 Odds Ratio (95% CI)	*p* Value
Female	T2 ^b^ vs. T1 ^b^	1.379 (1.013–1.877)	0.041	1.310 (0.958–1.792)	0.091	1.323 (0.967–1.810)	0.080
	T3 ^b^ vs. T1 ^b^	1.599 (1.171–2.184)	<0.003	1.522 (1.105–2.097)	0.010	1.539 (1.116–2.122)	0.008
Male	T2 ^b^ vs. T1 ^b^	1.232 (0.950–1.598)	0.116	1.192 (0.916–1.550)	0.191	1.208 (0.928–1.572)	0.160
	T3 ^b^ vs. T1 ^b^	1.207 (0.931–1.566)	0.155	1.038 (0.792–1.360)	0.787	1.046 (0.799–1.371)	0.742

^a^ Adjusted covariates: Model 1 = unadjusted; Model 2 = Model 1 + age, gender, percentage body fat, total cholesterol, uric acid, aspartate aminotransferase, creatinine, highly sensitive C-reactive protein, and thyroid-stimulating hormone; Model 3 = Model 2 + smoking and drinking. ^b^ Total calcium level: T1: 5–9.1 mg/dL, T2: 9.1–9.4 mg/dL, T3: 9.4–12 mg/dL.

**Table 5:** Gender difference in association between cut-off points of serum total calcium and the presence of high IOP.

Variables		Male	Female
**Cut-off value of serum total calcium (mg/dL)**		9.35	9.05
High IOP(>18 mmHg)	Model ^a^ 1 Odds Ratio (95% CI) *p* Value	1.258 (1.015–1.559) 0.036	1.764 (1.316–2.366) <0.001
	Model ^a^ 2 Odds Ratio (95% CI) *p* Value	1.129 (0.904–1.409) 0.285	1.689 (1.254–2.275) 0.001
	Model ^a^ 3 Odds Ratio (95% CI) *p* Value	1.136 (0.910–1.418) 0.261	1.704 (1.264–2.297) <0.001

^a^ Adjusted covariates: Model 1 = unadjusted; Model 2 = Model 1 + age, gender, percentage body fat, total cholesterol, uric acid, aspartate aminotransferase, creatinine, highly sensitive C-reactive protein, and thyroid-stimulating hormone; Model 3 = Model 2 + smoking and drinking.

## Data Availability

The data presented in this study are available on reasonable request from the corresponding author.
